# Scaffolding of a bacterial genome using MinION nanopore sequencing

**DOI:** 10.1038/srep11996

**Published:** 2015-07-07

**Authors:** E. Karlsson, A. Lärkeryd, A. Sjödin, M. Forsman, P. Stenberg

**Affiliations:** 1 Swedish Defence Research Agency, Umeå, Sweden; 2 Department of Chemistry, Computational Life Science Cluster (CLiC), Umeå University, Umeå, Sweden; 3 Molecular Biology, Umeå University, Umeå, Sweden

## Abstract

Second generation sequencing has revolutionized genomic studies. However, most genomes contain repeated DNA elements that are longer than the read lengths achievable with typical sequencers, so the genomic order of several generated contigs cannot be easily resolved. A new generation of sequencers offering substantially longer reads is emerging, notably the Pacific Biosciences (PacBio) RS II system and the MinION system, released in early 2014 by Oxford Nanopore Technologies through an early access program. The latter has highly advantageous portability and sequences samples by measuring changes in ionic current when single-stranded DNA molecules are translocated through nanopores. We show that the MinION system produces long reads with high mapability that can be used for scaffolding bacterial genomes, despite currently producing substantially higher error rates than PacBio reads. With further development we anticipate that MinION will be useful not only for assembling genomes, but also for rapid detection of organisms, potentially in the field.

Second generation sequencing platforms have revolutionised bacterial genomic research by giving many laboratories access to high-throughput and high quality sequencing. These platforms are capable of sequencing, in parallel, massive numbers of PCR-amplified DNA molecules. Most of them perform sequencing by synthesis, *i.e.* by imaging DNA polymerase-mediated nucleotide incorporation into growing DNA strands. They generate vast amounts of high quality sequence data but often suffer from amplification and sequencing bias complications, and their read lengths are generally restricted to a few hundred bases^1^,^2^.

These short read lengths make it difficult to obtain complete *de novo* assemblies for genomes that include longer repetitive sequences, so resolving the genomic order of some contiguous sequences (contigs) can be challenging. In our experience of sequencing and assembling *Francisella* genomes, sequencing with Illumina technology typically generates 20–100 contigs due to the presence of variable numbers of IS elements^3^.

However, a third generation of sequencers capable of producing reads several to tens of kilobases long is emerging. A prime example is the Pacific Biosciences (PacBio) RS II system, which has set the standard for commercial long-read sequencing by offering up to 30 kb reads. Like most other sequencing platforms it is based on sequencing by synthesis. However, it offers some major advantages over earlier technologies in that it can monitor individual DNA molecules, record the incorporation of fluorescently labelled nucleotides in real-time^4^, and eliminate much of the inherent sequencing bias of second generation platforms^1^. To improve sequence quality, in the PacBio system adapters are ligated at both ends of each DNA molecule to form a continuous DNA circle. This allows each DNA molecule to be sequenced through multiple passes to build a more accurate consensus read^5^. The technology has not yet matched second generation platforms in terms of sequence quality but has proven useful in various applications including contig scaffolding^6^.

Another promising platform is the MinION^TM^ device, which was released in early 2014 by Oxford Nanopore Technologies through an early access program (The MinION Access Program, MAP). Like the PacBio system, it delivers long read real-time sequencing of individual molecules. It also has two very distinctive features: it is the first commercial nanopore-based (rather than synthesis-based) sequencer, and is quite small and portable (measuring about 10 × 3 × 2 cm). MinION sequencing involves measuring the change in ionic current when a single-stranded DNA molecule is translocated through a protein nanopore in an insulated membrane^7^. The resulting current profile is then translated into sequence information in real time by a base calling software (Metrichor^TM^). Translocation is facilitated by ligating an adapter to one end of each DNA molecule with a bound enzyme that unwinds the double-stranded DNA and feeds a single strand through the pore. Provided that the DNA strand is kept intact during sample processing, there is no upper limit to the potential read length. A hairpin adapter ligated to the opposite end of the DNA molecule allows sequencing of both the sense and antisense strands, which are aligned to produce a higher quality consensus 2D read.

Here we present an update on the current status of the MinION sequencing system, and evaluate its utility for scaffolding bacterial genomes by comparing MinION sequencing data for two *Francisella* genomes to corresponding data acquired using the PacBio RS II system.

## Results

### Reference genome

To evaluate the MinION sequencing system it was used to sequence the bacterial FSC1006 (CP009574) genome using the R7 flow cell and the FSC996 (CP010427) genome using the newer R7.3 flow cell. The results obtained were compared to corresponding data acquired using the Pacific Biosciences RS II system. In addition, the genomes were sequenced using an Illumina HiSeq machine. The circular chromosomes of FSC1006 and FSC996 are 2015987 and 1658482 base pairs long, respectively. Their average GC contents are 32.4% (FSC1006) and 32.0% (FSC996), but both genomes contain a ~5 kb triplicated region with an average GC content of 45.7% (FSC1006) or 47.8% (FSC996). In addition, short tandem repeats (one to nine bp) with copy numbers of at least five constitute around 5% of each genome. These features were used to assess overall sequence quality and the effect of variation in genome sequence composition on sequence quality for each of the tested sequencing technologies. All of the reported results are based on the sequencing of the FSC996 genome using the newer (R7.3) MinION flow cell unless stated otherwise. Sequencing results for the FSC1006 genome are used to compare the R7 and R7.3 MinION flow cells.

### Amount and length of reads

**Figure 1:**
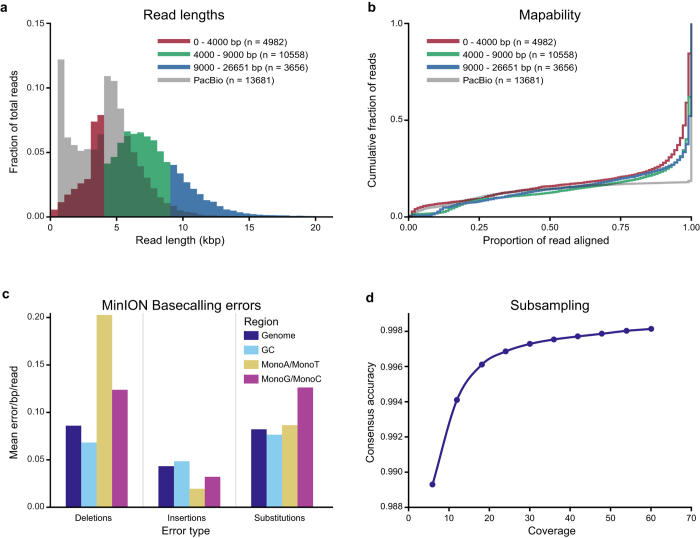
Quality of MinION (R7.3) and PacBio sequencing reads. (**a**) Length distribution of the reads. MinION reads are divided into three length categories that are coloured separately. Note that the high number of MinION reads of about 3.5 kb originate from the ligation control fragment. (**b**) Mapability of PacBio and MinION reads divided into the same length categories as in (**a**). Read alignment length is the fraction of the reads covered in the BLAST alignment against the reference genome. (**c**) Mean frequencies of deletion, insertion and substitution errors per nucleotide per read for MinION sequence reads in four genomic regions: whole genome (32% GC), high GC-content regions (47.8% GC), A/T and G/C monomers at least 5 bp long. (**d**) Consensus accuracy versus average read coverage of the genome. Different coverages were obtained by subsampling the reads from a single MinION run.

The MinION run produced 61236 sequencing reads of which 19196 (31.3%) were classified as “2D pass” and 11227 (18.3%) as “2D fail” by the Metrichor^TM^ Agent. The remainder consisted of template or complement-only reads. The mean and maximum lengths of the 2D pass reads were 6402 and 26651 base pairs, respectively (for comparisons with the results obtained using the R7 cell, see Supplementary Table S1). The complete read length distribution is shown in Fig. 1A; note that the peak at 3.5 kb originates from a 3560 bp ligation control added during library preparation. For a comparison of the results obtained using the R7.3 and R7 cells, see Supplementary Figure S1.

The PacBio RS II sequencing was done using two SMRT Cells and yielded 90148 reads with 13681 corrected reads using the RS_PreAssembler.2 protocol in the SMRT Analysis Software. The read length distribution of this PacBio sequencing run is also shown in Fig. 1A. The Illumina sequencing generated 17862240 paired-end reads (100 bp).

### Mapping reads to the reference genome

The quality of reads generated by the MinION and the PacBio systems was investigated by aligning them against the complete FSC996 reference genome using BLAST. We started by evaluating which parameter settings produced the best alignments (see Supplementary Figure S2). More stringent parameters produce alignments with fewer indels and mismatches but lower genome coverage (calculated as the summed aligned length of all reads divided by the genome size) because larger parts of the reads cannot be aligned. Since we wanted to use the reads for scaffolding we prioritized high coverage (*i.e.* long alignments). We decided to use gap opening and gap extension penalties of 2 and match/mismatch scores of 2 and −3, respectively, as these were the most stringent parameter settings that could be used while retaining high total coverage (see Supplementary Figure S2). The MinION reads were aligned against the ligation control DNA fragment as well as the FSC996 reference genome. Hits against one of the two reference sequences were obtained for 19174 (99.9%) of the MinION 2D pass reads. To remove bias that may have been created by the presence of several repeated regions, only the best hit for each read was considered in subsequent analyses. In addition, 2066 MinION reads for which the best hits were in the control DNA sequence were excluded to avoid inter-species bias, leaving 17108 alignments.

The proportions of the reads’ lengths covered by these alignments are shown in Fig. 1B. Alignments for 2.6% of the reads covered less than 10% of their length, but most (70.2%) covered at least 95% of the corresponding reads. Short reads (<4000 bp) had slightly poorer quality than medium-sized and long ones, indicating that the proportional alignment length increased with the read length (Fig. 1C). Alignments of the PacBio sequence reads provided greater proportional alignment lengths; 82.3% of the PacBio reads aligned with at least 95% of the corresponding read length.

### Base calling errors

To investigate error rates in the final sequence reads generated by the two systems, rates of insertion, deletion and substitution errors in each case were evaluated by analysing the BLAST alignments against the FSC996 reference sequence. It should be noted that (as can be seen in Supplementary Figure S2) error rates vary with BLAST parameters. The rates of all three error types differed substantially both within and between the sets of MinION and PacBio sequences (Fig. 1C and Supplementary Figure S3). Insertion errors were the least frequent, with substitution and deletion errors occurring at very similar rates in the MinION reads. In contrast, for the PacBio reads deletions were more common than insertions, which in turn were more common than mismatches. However, the frequencies of different error types in the MinION reads were 25 to 160-fold higher than in the PacBio reads, even though the error frequencies achieved with the R7.3 MinION cell were 15–40% lower than those achieved with the older R7 cell (Supplementary Figure S3). To assess the variation in read quality between individual reads, we plotted the indel and mismatch frequencies (as reported by BLAST) of all “2D pass” and “2D fail” reads producing at least 2 kb long alignments to the reference genome (Fig. 1D). The “2D pass” reads had fewer errors but several of the “2D fail” reads could potentially be used in certain downstream analyses.

**Figure 2:**
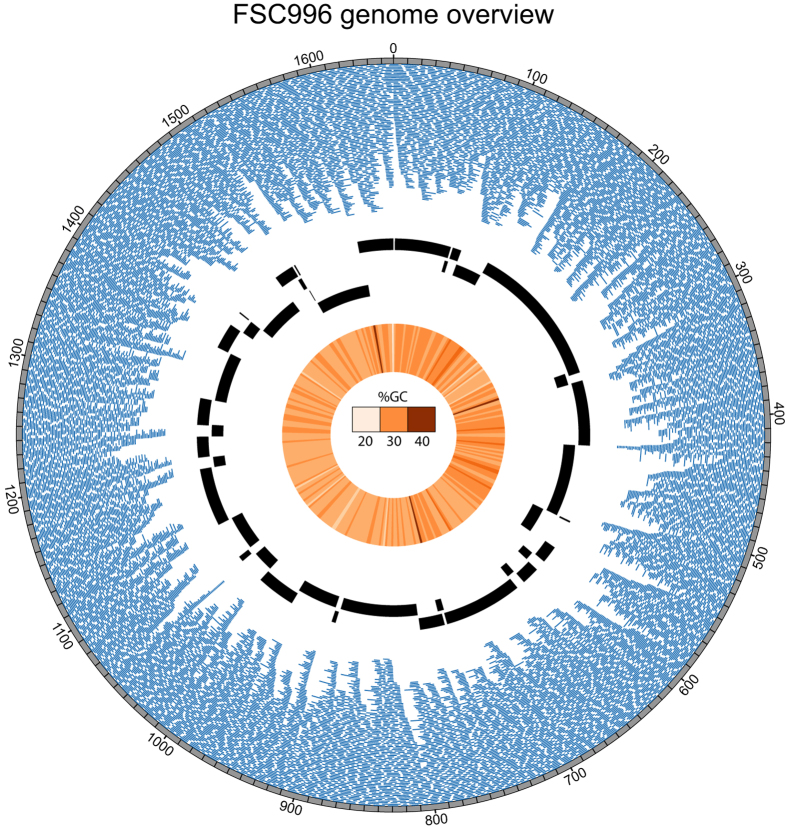
Overview of the FSC996 reference genome and the MinION data. The outermost track represents the reference genome with genomic positions (in kb). The blue lines provide a visual representation of every MinION read longer than 2 kb used in the analysis and its genomic alignment position (tile layers limited to 100). The black lines show the contigs generated from the assembly of Illumina Hiseq reads and their alignment positions. The inner circle shows a heat map of the GC content of the reference genome, with the brightest colour representing 20% GC and the darkest 40%.

To assess the potential effects of variation in the GC content on error rates we compared the error rates for reads in the triplicated region of the FSC996 genome, which has a relatively high GC-content as shown in Fig. 2, to those for the rest of the genome. The GC content was found to have minor effects on the error rates of reads (Fig. 1C). To see if simple sequence repeats had any effects on error rates, we mapped all 1- to 10-nucleotide long sequences present in at least five copies in the FSC996 genome. Most of these repeats consisted of monomers (n = 16269) with lengths of 5–10 bp. We also found 30 di-nucleotide repeats (mostly AT repeats) and four nine bp repeats at least five units long. The frequency of deletion errors in monomers of A or T was more than twice that for the genome as a whole (the error frequencies for A and T monomers were very similar for all error types, so they were analysed as a single group) whereas the frequency of insertion errors in A/T monomers was around half that for the whole genome (Fig. 1C). Monomers of G and C also exhibited very similar error frequencies for all error types. Like A/T monomers, G/C monomers exhibited a somewhat higher frequency of deletions and a lower frequency of insertions than the genome as a whole, but these differences were less pronounced than those observed for A/T monomers. Conversely, whereas the frequency of mismatch errors among A/T monomers was virtually identical to the value for the whole genome, that for G/C monomers was substantially higher. Repeat units of two and nine bp appeared to have higher frequencies of deletion errors than other repeat types, although this conclusion is based on relatively few observations (Supplementary Figure S5). Overall, we conclude that while the R7.3 version of the MinION system produces lower error frequencies (by around 15–40%) than the R7 system, its error rates remain substantially higher than those achieved with the PacBio system. It should be noted that this conclusion has not been verified in genomes with high GC contents; the FSC996 genome has a relatively low GC-content of 32%.

We next sought to determine whether the high error rates of the MinION system would render its reads unusable for variant calling. After subsampling the reads to create a range of different genome coverage levels, we mapped them back to the reference genome and kept the most common nucleotide at each position. In our case, sequencing a genome of 1.66 Mbp on a single MinION flow cell yielded a coverage of 60 and a consensus accuracy of 99.8%. Fig. 1D shows how coverage affects consensus accuracy. For comparative purposes, a single PacBio sequencing run produced a coverage of 29 and a consensus accuracy of 99.998%.

### Scaffolding using MinION reads

The scaffolding capabilities of the MinION sequence reads were tested by scaffolding contigs produced by assembling Illumina reads (sequenced to ~1000X coverage) using ABySS 1.5.2 with default parameters. The Illumina *de novo* assembly produced 40 contigs (representing parts of the main chromosome) with an N50 of 76819, as shown in Fig. 2. SSPACE-LongRead was then used together with BLASR to scaffold the contigs using the MinION reads (see *Materials and Methods*). The MinION reads (shown aligned to the genome in Fig. 2) could correctly join all of the contigs to a complete chromosome. 1171 reads supported correct links between contigs and 6 supported incorrect links. We then tried to scaffold using the subsampled reads (described above); in this case, only 20% of the reads were required to correctly join all of the contigs. The PacBio reads could also correctly scaffold the chromosome and proved to be sufficient for *de novo* assembly of the full chromosome using the SMRT Analysis software. Recently, Loman *et al.*^8^ and Goodwin *et al.*^9^ made available pipelines for *de novo* assemblies and hybrid assemblies respectively using MinION reads. We used the former to *de novo* assemble the FSC996 chromosome which resulted in two contigs. These two contigs could subsequently be joined to a complete chromosome using SSPACE. Although, the above pipelines are in early stages of development, MinION reads can clearly be used also for *de novo* assemblies. We conclude that the MinION system is a viable alternative to the PacBio system for scaffolding genomes whether they have already been sequenced by *e.g.* Illumina or not.

## Discussion

We here present an update on the performance of the MinION third generation sequencer. In our hands, the MinION system generated an average read accuracy of 79% (similar to that reported by Quick *et al.*^10^) when sequencing the AT-rich FSC996 genome using R7.3 chemistry. For comparative purposes, the PacBio RS II system gave, for the same genome, an average read accuracy of 86.4% for the raw reads and 99.5% after the SMRT PreAssembler.2 error correction. Despite the relatively high error rate, our results indicate that MinION reads are valuable for scaffolding bacterial genomes due to their length and uniform mapability.

The PacBio RS II platform represents a challenging benchmark because it has been marketed for several years (whereas the MinION system is still being developed and has not yet been fully commercialised) and is the only other system that offers similar read lengths. It should be noted that the read lengths obtained in this study do not demonstrate either technique’s full potential. If necessary, both the MinION and PacBio read lengths can be raised by increasing the input DNA fragment sizes, and several studies have reported PacBio reads of over 20–30 kb^11^,^12^,^13^. The longest MinION 2D read generated in this study exceeded 30 kb (34 kb FSC996, 31 kb FSC1006), and as the Nanopore sequencing technology is not synthesis-based it seems to have fewer inherent limitations in terms of potential read length. Furthermore, long MinION reads had higher sequence quality (in terms of the proportion of their lengths that fully aligned with the reference sequence) than short reads.

The average error rate per nucleotide in the MinION reads was 0.2, with deletions and substitutions being around twice as common as insertions. However, higher error rates were observed for simple sequence repeats, especially A/T mononucleotide repeats but also G/C mononucleotide repeats to a lesser extent. This is somewhat inconsistent with the results reported by Ashton *et al.*^14^, who observed the highest deletion error rates in G/C mononucleotide repeats. This discrepancy could be due to the fact that Ashton *et al.*^14^ looked at repeats that were up to six base pairs long whereas we focused on repeats with five or more repeat units. In addition, Ashton *et al.* used the R7 flow cell and a different mapping method. It should also be noted that estimated error rates are dependent on the choice of aligner as well as alignment parameters (see Supplementary Figure S2). We selected alignment parameters with the aim of maximizing the aligned fraction of each read while retaining relatively high stringency. Despite the high overall error rate, the achieved genome coverage of 60 was sufficient to obtain a consensus accuracy of 99.8% when the reads were mapped back to the reference genome. The FSC996 genome has a lower GC content (32%) than most bacterial genomes, even in its most GC-rich regions (47.8%), so the MinION system’s ability to sequence extremely GC-rich sequences requires further evaluation.

The PacBio system also has higher error rates than second generation platforms (albeit orders of magnitudes lower than those for the MinION reads), but the error rates in its reads increase with fragment size^15^ due to corresponding reductions in the number of possible full passes of the inserts. Thus, both the MinION and PacBio systems currently provide useful complements to second generation sequencing platforms, which afford higher coverage and lower error rates but much shorter sequencing reads. However, as the third generation sequencers improve they may become viable stand-alone competitors. Notably, the MinION library preparation as well as other processes are being continuously improved to reduce handling times and increase numbers of 2D reads. During the few months in which we have participated in the MinION access program we have already seen rapid improvements in sequence quality, and we expect coming upgrades to deliver further progress. The chemistry employed in the PacBio sequencing system has also improved since we sequenced the FSC996 and the FSC1006 genomes.

Attempts to assemble bacterial genomes from short read sequences commonly yield several contigs that are hard to piece together. Our attempt to construct a *de novo* assembly of the FSC996 chromosome based on Illumina sequence data (sequenced to ~1000X coverage) produced 40 contigs that were readily organized into a complete chromosome using either the MinION or the PacBio reads. In fact, only 20% of the reads from a single MinION run were needed to correctly scaffold the genome. Thus, the MinION system is very suitable for closing genomes that have previously been sequenced with short reads. Ideally, only a single sequencing run would be needed to produce a complete chromosome. Indeed, by using either the PacBio or MinION reads, we were able to accurately *de novo* assemble the chromosome. However, with current error rates and a genome the size of FSC996, a single MinION run produce a genome with about 99.8% sequence accuracy.

Although the MinION and PacBio systems provide reads of similar length, we envisage them to have slightly different target users and applications. PacBio platforms are large, heavy, initially costly and primarily suitable for sequencing centres with extensive supporting infrastructure, but they offer relatively low operating costs per sample. In contrast, the MinION system is packaged in a convenient portable device that will require a much smaller initial investment and will thus be more accessible for smaller laboratories. The small size of the MinION system means that it could potentially offer portable real-time sequencing capabilities, which would enable much more general analysis of samples than is possible with real-time PCR and would be an extremely attractive feature. Specifically, it would enable continuous scanning of reads for sequence information related to factors such as pathogenicity and antibiotic resistance. If the system’s requirements in terms of hands-on time and quantity of input material could be reduced, this would be particularly advantageous for diagnostic purposes.

## Methods

### Sample preparation

Genomic DNA from two strains of Francisella, FSC996 and FSC1006^16^, were extracted using a previously described phenol/chloroform method^17^. The isolated DNA was used for sequencing with a MinION system (Oxford Nanopore Technologies Ltd [ONT], Oxford, UK) in-house. DNA preparations were also sent for sequencing by a Pacific Biosciences RS II system (10 kb library, P4-C2 chemistry, 120 min data collection) at the Uppsala Genome Center (Uppsala, Sweden) and for Illumina HiSeq 2000 sequencing (100 bp, paired end) at SNP&SEQ (Uppsala, Sweden). All sequencing operations were performed using the reagents and protocols recommended by the manufacturers.

### MinION library preparation and sequencing of FSC996 (R7.3 chemistry)

To remove any small DNA fragments that might have formed during the phenol/chloroform extraction procedure, high molecular weight DNA was isolated by gel extraction after being electrophoresed on a 0.8% agarose gel with 1 × GelRed (Biotium, Hayward, CA, USA) in 1 × TEA buffer. The DNA was visualized on a blue light transilluminator (Dark Reader Transilluminator, Clare Chemical Research, Dolores, CO, USA) and DNA fragments larger than 12 kbp (identified by comparison to a 1 kb Plus DNA Ladder, Life Technologies, Carlsbad, CA, USA) were excised from the agarose gel using a scalpel. The excised DNA was then purified using the Zymoclean Large Fragment DNA Recovery Kit (Zymo Research, Irvine, CA, USA). The amount of recovered DNA was determined using a Qubit fluorometer (Life Technologies) and 1.5 μg of the DNA (in 80 μl nuclease-free water, Qiagen, Hilden, Germany) was fragmented by g-TUBE centrifugation at 2300 g for 2 × 60 s (Covaris Inc., Woburn, MA, USA). The DNA was then subjected to 30 min of PreCR treatment at 37 °C (in 100 μl PreCR Repair Mix, New England Biolabs [NEB], Ipswich, MA, USA), followed by DNA clean-up using 1 × volume of Agencourt AMPure XP beads (Beckman-Coulter, Brea, CA, USA) according the manufacturer’s protocol, except that 80% ethanol was used for washing. The DNA was eluted in 80 μl nuclease-free water (Qiagen). After clean-up, 50 ng of ligation control DNA was added to the DNA sample (DNA CS, 3560 bp, provided by ONT). The fragmented DNA was then end-repaired using the NEBNext® End Repair Module (NEB) for 30 min at room temperature, followed by Agencourt AMPure XP (Beckman-Coulter) clean-up using 1 × volume of beads (80% ethanol wash, 25 μl elution volume). The end-repaired DNA was then dA-tailed using the NEBNext® dA-Tailing Module (NEB) for 30 min at 37 °C (30 μl reaction volume).

The library for FSC996 was further prepared using the SQK-MAP003 genomic sequencing kit with replacement wash and elution buffers for His-tag bead clean-up as provided by ONT. The dA-tailed DNA (30 μl) was mixed with 8 μl nuclease-free water, 10 μl adapter mix, 2 μl HP adapter and 50 μl Blunt/TA Ligase Master Mix (NEB) and incubated for 10 min at room temperature in a Protein LoBind microcentrifuge tube (Eppendorf, Hamburg, Germany). The adapter-ligated DNA was then purified with His-tag beads (Dynabeads His-Tag Isolation and Pulldown, Life technologies). The His-tag beads (10 μl) were washed twice in 1 × wash buffer, using a magnetic rack to pellet the beads, and then resuspended in 100 μl of 2 × wash buffer. The resulting suspension was carefully mixed with the 100 μl ligation reaction mixture, incubated for 5 min at room temperature, and then placed on a magnetic rack, after which the beads were washed twice with 250 μl 1 × wash buffer. The wash buffer was aspirated according to the recommendations in the sequencing kit protocol, and the library was eluted in 25 μl elution buffer.

Prior to sequencing, an R7.3 MinION flow cell (FLO-MAP003) was primed with 2 × 150 μl EP buffer, with a 10 min incubation period after each addition. A 6 μl portion of the FSC996 library, containing 43.3 ng DNA as measured using a Qubit fluorometer (Life Technologies), was diluted in 140 μl EP buffer and 4 μl fuel mix and then loaded into the primed flow cell. Sequencing was performed for 48 hours, with reloading of the library (in appropriate volumes of EP buffer and fuel mix) after 16 (43.3 ng), 24 (43.3 ng) and 40 hours (21.7 ng). In total 151.6 ng of library DNA was loaded into the flow cell.

### MinION library preparation and sequencing of FSC1006 (R7 chemistry)

For the FSC1006 strain, 2.0 μg (150 μl) of the phenol/chloroform extracted DNA was fragmented using a g-TUBE (Covaris) at 3300 g for 2 × 60 s. 50 ng of ligation control (DNA CS) was added to 1.0 μg of the fragmented DNA followed by end-repair and dA-tailing as described above. The library was further prepared using the SQK-MAP002 (ONT) genomic sequencing kit. 10 μl of each adapter mix and HP adapter was added to the dA-tailed DNA (30 μl) and mixed with 50 μl Blunt/TA Ligase Master Mix (NEB). The ligation reaction was incubated for 10 min at room temperature in a DNA LoBind microcentrifuge tube (Eppendorf) after which the ligated fragments were purified with 0.4 × Agencourt AMPure XP beads (Beckman-Coulter) using the wash and elution buffers provided with the sequencing kit. The beads were washed once in 150 μl wash buffer and eluted in 25 μl of elution buffer. Tethers (10 μl) and HP motor (15 μl) were added sequentially to the eluted DNA and incubated for 10 min and 3 hours at room temperature, respectively (these incubation steps have been removed in the protocol supplied with the SQK-MAP003 sequencing kit).

An R7 MinION flow cell (FLO-MAP002), was primed as described above. 21 ng (6 μl) of the FSC1006 library (DNA loading determined with a Qubit fluorometer) was mixed with 140 μl EP buffer and 4 μl fuel mix, and loaded into the primed flow cell. The library was sequenced for 72 hours, with fresh library DNA being added after 17 (21 ng), 24 (21 ng), 41 (42 ng), and 64 hours (21 ng) using appropriate volumes of EP buffer and fuel mix. In total 126 ng of library DNA was loaded into the flow cell.

### Base calling and read processing

The raw MinION sequence data were base called using the Metrichor^TM^ Agent. The base calling workflows *R7.X 2D Basecalling rev 1.9* and *R7 2D workflow rev 1.4* were used for the FSC996 and FSC1006 data, respectively. The base called sequencing reads were retrieved in fast5-format and, using Metrichor^TM^ Agent (*rev 1.9*), automatically sorted into two folders - one containing passed full 2D reads and the other containing failed 2D reads below the quality threshold, 1D template reads and 1D complement reads. PacBio sequence reads were retrieved using the RS_PreAssembler.2 protocol in SMRTAnalysis v2.2.0.p3.

### BLAST alignment

The BLAST alignments were conducted using blastn version 2.2.27 + with the following parameters: word size = 11, reward = 2, penalty = −3, gapopen = 2 and gapextend = 2^18^.

### Scaffolding

The SSPACE-LongRead perl script was used to scaffold contigs with MinION data. SSPACE-LongRead uses BLASR to perform alignments^6^,^19^. The specific arguments resulting in one correctly assembled chromosome in SSPACE-LongRead were i = 70, a = 1500 and g = −5000 for the MinION reads and i = 90, a = 1500 and g = −7000 for the PacBio reads.

### Subsampling of MinION reads

The MinION sequence reads were randomly subsampled to 10 overlapping levels (10, 20, 30, etc. percent of total reads) to ensure that performance differences between coverage levels were due to added information as opposed to sampling a different set of sequence reads.

### Consensus sequence

LAST^20^ was used to align the sequence reads against the reference using parameters q = 1 and a = 1 after which the alignments were converted into SAM format. A consensus sequence was created using these data by selecting the most common nucleotide in each position. The consensus sequences were then aligned against the reference using BLAST with default parameters to determine the number of mismatching base pairs.

## Additional Information

**Accession codes:** MinION, PacBio and Illumina raw data, as well as reference genomes are available for download from the NCBI (FSC1006; BioProject: PRJNA261387, FSC996; BioProject: PRJNA271279).

**How to cite this article**: Karlsson, E. *et al.* Scaffolding of a bacterial genome using MinION nanopore sequencing. *Sci. Rep.* **5**, 11996; doi: 10.1038/srep11996 (2015).

## Supplementary Material

Supplementary Information

## References

[b1] Ross M. G. *et al.* Characterizing and measuring bias in sequence data. Genome Biol 14, R51, 10.1186/gb-2013-14-5-r51 (2013). 23718773 PMC4053816

[b2] Loman N. J. *et al.* Performance comparison of benchtop high-throughput sequencing platforms. Nat Biotechnol 30, 434–439, 10.1038/nbt.2198 (2012). 22522955

[b3] Sjödin A. *et al.* Genome characterisation of the genus Francisella reveals insight into similar evolutionary paths in pathogens of mammals and fish. BMC Genomics 13, 268, 10.1186/1471-2164-13-268 (2012). 22727144 PMC3485624

[b4] Eid J. *et al.* Real-time DNA sequencing from single polymerase molecules. Science 323, 133–138, 10.1126/science.1162986 (2009). 19023044

[b5] Travers K. J., Chin C. S., Rank D. R., Eid J. S. & Turner S. W. A flexible and efficient template format for circular consensus sequencing and SNP detection. Nucleic Acids Res 38, e159, 10.1093/nar/gkq543 (2010). 20571086 PMC2926623

[b6] Boetzer M. & Pirovano W. SSPACE-LongRead: scaffolding bacterial draft genomes using long read sequence information. BMC Bioinformatics 15, 211, 10.1186/1471-2105-15-211 (2014). 24950923 PMC4076250

[b7] Schneider G. F. & Dekker C. DNA sequencing with nanopores. Nat Biotechnol 30, 326–328, 10.1038/nbt.2181 (2012). 22491281

[b8] Loman N. J., Quick J. & Simpson J. T. A complete bacterial genome assembled *de novo* using only nanopore sequencing data. bioRxiv., 10.1101/015552 (2015). 26076426

[b9] Goodwin S. *et al.* Oxford Nanopore Sequencing and *de novo* Assembly of a Eukaryotic Genome. bioRxiv., 10.1101/013490 (2015). PMC4617970 26447147

[b10] Quick J., Quinlan A. R. & Loman N. J. A reference bacterial genome dataset generated on the MinION™ portable single-molecule nanopore sequencer. Gigascience 3, 22, 10.1186/2047-217X-3-22 (2014). 25386338 PMC4226419

[b11] Parker D. *et al.* Genome Sequence of Bacterial Interference Strain Staphylococcus aureus 502A. Genome Announc 2, 10.1128/genomeA.00284-14 (2014). PMC3983310 24723721

[b12] Brown S. D. *et al.* Complete Genome Sequence of Pelosinus sp. Strain UFO1 Assembled Using Single-Molecule Real-Time DNA Sequencing Technology. Genome Announc 2, 10.1128/genomeA.00881-14 (2014). PMC4155594 25189589

[b13] Terabayashi Y. *et al.* First complete genome sequence of*Salmonella enterica* subsp. *enterica* serovar Typhimurium strain ATCC 13311 (NCTC 74), a reference strain of multidrug resistance, as achieved by use of PacBio single-molecule real-time technology. Genome Announc 2, 10.1128/genomeA.00986-14 (2014). PMC4183876 25278532

[b14] Ashton P. M. *et al.* MinION nanopore sequencing identifies the position and structure of a bacterial antibiotic resistance island. Nat Biotechnol., 10.1038/nbt.3103 (2014). 25485618

[b15] Jiao X. *et al.* A Benchmark Study on Error Assessment and Quality Control of CCS Reads Derived from the PacBio RS. J Data Mining Genomics Proteomics 4, 10.4172/2153-0602.1000136 (2013). PMC3811116 24179701

[b16] Sjödin A. *et al.* Complete Genome Sequence of Francisella endociliophora Strain FSC1006, Isolated from a Laboratory Culture of the Marine Ciliate Euplotes raikovi. Genome Announc 2, 10.1128/genomeA.01227-14 (2014). PMC4246165 25428973

[b17] Larsson P. *et al.* Molecular evolutionary consequences of niche restriction in Francisella tularensis, a facultative intracellular pathogen. PLoS Pathog 5, e1000472, 10.1371/journal.ppat.1000472 (2009). 19521508 PMC2688086

[b18] Altschul S. F., Gish W., Miller W., Myers E. W. & Lipman D. J. Basic local alignment search tool. J Mol Biol 215, 403–410, 10.1016/S0022-2836(05)80360-2 (1990). 2231712

[b19] Chaisson M. J. & Tesler G. Mapping single molecule sequencing reads using basic local alignment with successive refinement (BLASR): application and theory. BMC Bioinformatics 13, 238, 10.1186/1471-2105-13-238 (2012). 22988817 PMC3572422

[b20] Frith M. C., Hamada M. & Paul H. Parameters for accurate genome alignment. BMC Bioinformatics. 11, 80, 10.1186/1471-2105-11-80 (2010). 20144198 PMC2829014

