# Supplementary material for: Scaffolding of a bacterial genome using MinION nanopore sequencing
Source: Sci Rep. 2015 Jul 7;5:11996. doi: 10.1038/srep11996 (PMC4493687; doi:10.1038/srep11996)
Supplement: Supplementary Information [file srep11996-s1.pdf]

Supplementary Information

Scaffolding of a bacterial genome using MinION nanopore sequencing

Karlsson, E. [1§], Lärkeryd, A. [1§], Sjödin, A. [1,2], Forsman, M. [1] and Stenberg, P. [1,2,3*]

[1]Swedish Defence Research Agency, Umeå, Sweden
[2]Department of Chemistry, Computational Life Science Cluster (CLiC), Umeå University, Umeå, Sweden
[3]Molecular Biology, Umeå University, Umeå, Sweden
[§]Equal contribution
[*]Correspondence: Per.Stenberg@foi.se

Supplementary Tables

Sequencing run	Number of 2D reads	Number of bases	Mean length	Median length	Max length	Mapped bases
R7.3 (FSC996)	30423	190148225	6250	6038	34370	128164261
R7 (FSC1006)	20099	117183952	5830	5580	30578	82872274

Supplementary Table S1. Sequence output (all 2D reads) from the MinION R7 (FSC1006 genome) and the R7.3 (FSC996 genome) sequencing runs.

Supplementary Figures

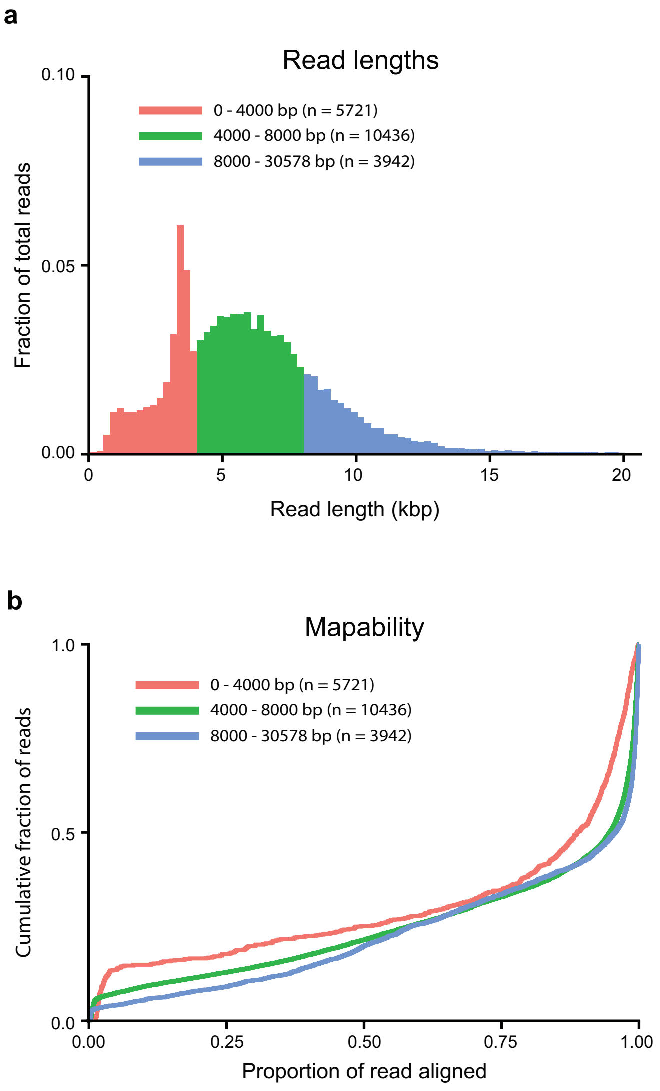

Supplementary Figure S1. Quality of MinION (R7) sequencing reads from FSC1006. (a) Length distribution of the reads. MinION reads are divided into three length categories that are coloured separately. Note that the high number of MinION reads of about 3.5 kb originate from the ligation control fragment. (b) Mapability of MinION reads divided into the same length categories as in (a). Read alignment length is the fraction of the reads covered in the BLAST alignment against the reference genome.

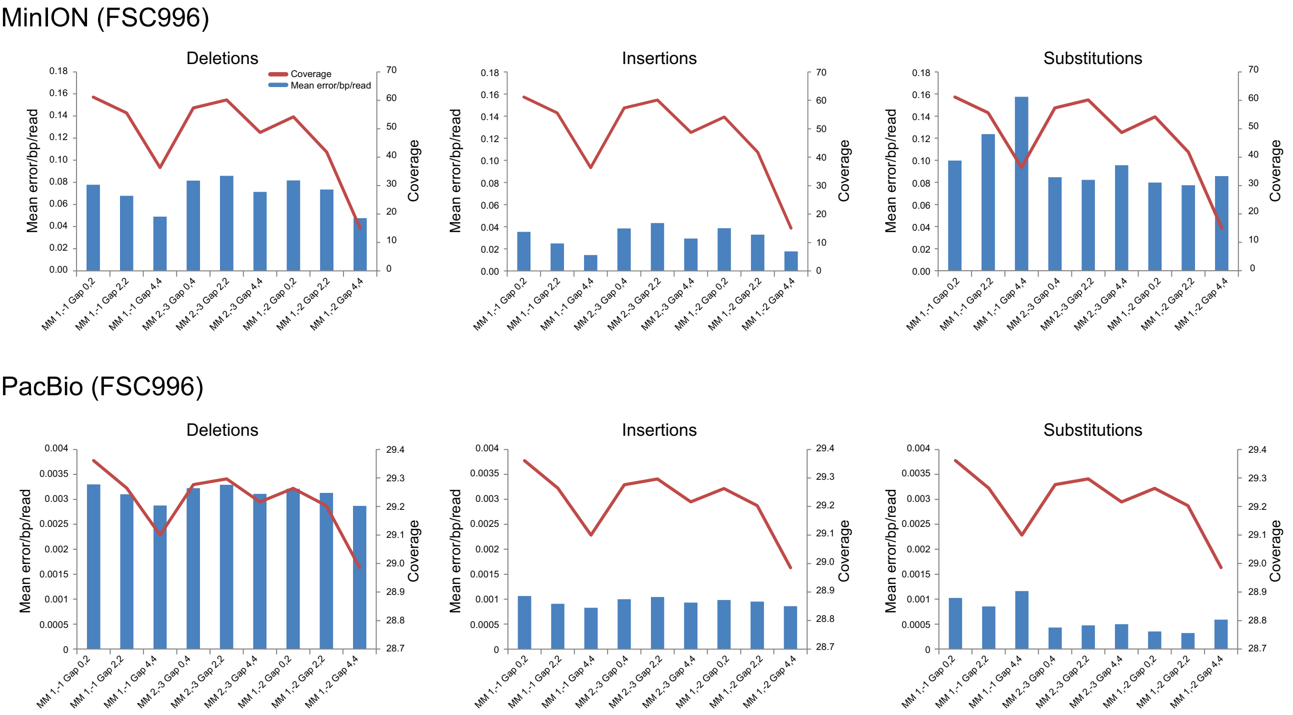

Supplementary Figure S2. Error rates and genomic coverage both vary with BLAST parameters. Mean error rates (deletions, insertions and substitutions) per base pair per read and genomic coverage (calculated as the summed aligned length of all reads divided by the genome size) after mapping MinION (R7.3) and PacBio reads to the FSC996 reference genome using different BLAST parameters. MM=match and mismatch scores and Gap=gap opening and gap extension penalties. Note that for match and mismatch scores of 2 and -3 respectively, a gap opening penalty of 0 combined with a gap extension penalty of 2 is not allowed by BLAST. Therefore a gap extension penalty of 4 was used instead.

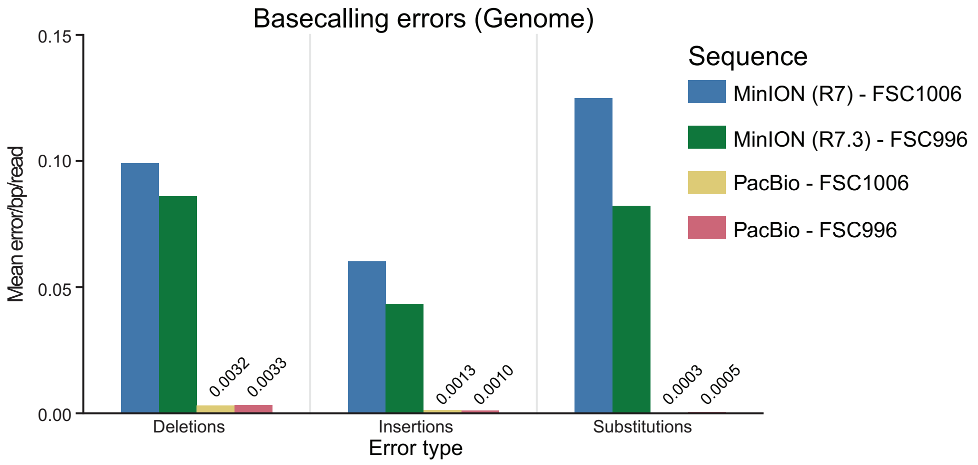

Supplementary Figure S3. Error rates in the sequence reads generated by the two MinION (R7 and R7.3) and PacBio runs. Mean error rates (deletions, insertions and substitutions) per base pair per read across the FSC996 and FSC1006 genomes are shown.

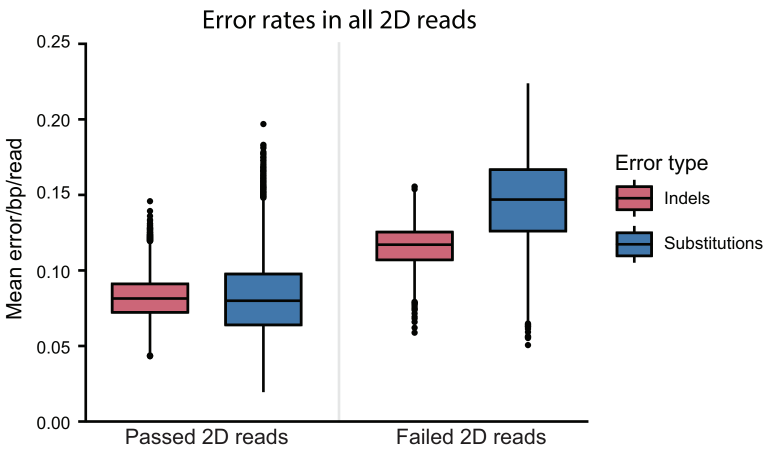

Supplementary Figure S4. Boxplot showing the difference in rates of Indels and substitutions between 2D MinION reads (R7.3) that passed and failed quality filtering. Thick black lines and boxes indicate median values and the 25th to 75th quartile range, respectively. Whiskers represent 1.5x the inter-quartile range and black dots denote outliers.

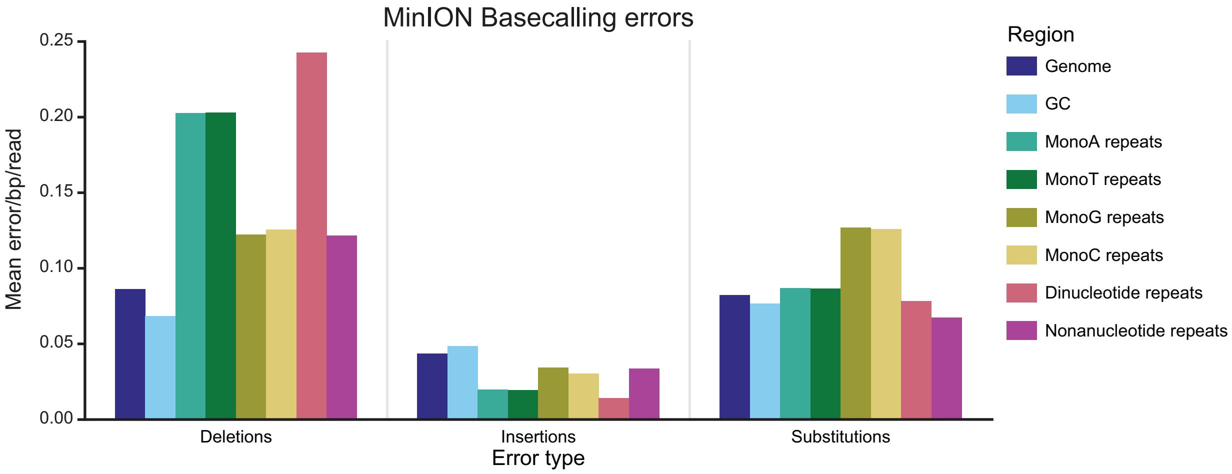

Supplementary Figure S5. Error rates within different genomic regions in the sequence reads generated by MinION (R7.3) sequencing. Mean error rates (deletions, insertions and substitutions) per base pair per read in the genome (32% GC), high GC-regions (47.8% GC), monomer repeats (A, T, G and C), dimer repeats and nonamer repeats. All repeats are at least five repeat units long.
